# Late-Stage Glioma Is Associated with Deleterious Alteration of Gut Bacterial Metabolites in Mice

**DOI:** 10.3390/metabo12040290

**Published:** 2022-03-25

**Authors:** Aglae Herbreteau, Philippe Aubert, Mikaël Croyal, Philippe Naveilhan, Stéphanie Billon-Crossouard, Michel Neunlist, Yves Delneste, Dominique Couez, Laetitia Aymeric

**Affiliations:** 1 University of Angers, Nantes Université, CHU Angers, Inserm, CNRS, CRCI2NA, SFR ICAT, F-49000 Angers, France; aglae.herbreteau@univ-angers.fr (A.H.); yves.delneste@univ-angers.fr (Y.D.); dominique.couez@univ-angers.fr (D.C.); 2 Nantes Université, Inserm, TENS, The Enteric Nervous System in Gut and Brain Disorders, IMAD, F-44000 Nantes, France; philippe.aubert@univ-nantes.fr (P.A.); philippe.naveilhan@univ-nantes.fr (P.N.); michel.neunlist@inserm.fr (M.N.); 3 CHU Nantes, Université de Nantes, CNRS, INSERM, L’Institut du Thorax, F-44000 Nantes, France; mikael.croyal@univ-nantes.fr (M.C.); stephanie.crossouard@univ-nantes.fr (S.B.-C.); 4 CHU Nantes, Université de Nantes, CNRS, SFR Santé, Inserm UMS 016, CNRS UMS 3556, F-44000 Nantes, France; 5 CRNH-Ouest Mass Spectrometry Core Facility, F-44000 Nantes, France

**Keywords:** gut microbiota, bacterial metabolites, late-stage glioma, immune environment

## Abstract

Brain-gut axis refers to the bidirectional functional connection between the brain and the gut, which sustains vital functions for vertebrates. This connection also underlies the gastrointestinal (GI) comorbidities associated with brain disorders. Using a mouse model of glioma, based on the orthotopic injection of GL261 cell line in syngeneic C57BL6 mice, we show that late-stage glioma is associated with GI functional alteration and with a shift in the level of some bacterial metabolites in the cecum. By performing cecal content transfer experiments, we further show that cancer-associated alteration in cecal metabolites is involved in end-stage disease progression. Antibiotic treatment results in a slight but significant delay in mice death and a shift in the proportion of myeloid cells in the brain tumor environment. This work rationally considers microbiota modulating strategies in the clinical management of patients with late-stage glioma.

## 1. Introduction

The gut microbiota and particularly bacterial metabolites are important players in the gut/brain functional bi-directional connection, the so-called “brain-gut axis” [1]. Specific bacterial metabolites, including short-chain fatty acids (SCFA) produced by colonic fiber fermentation [2] and secondary bile acids [3], have raised particular interest in the field due to their association with health and their involvement in different disorders, including brain diseases [1].

Bacterial metabolites produced in the lumen directly or indirectly signal to the brain through the humoral or neuronal route via the vagus nerve, a major anatomic connection between the gut and the brain. Hence, SCFA strengthen the blood-brain barrier during development, thus reducing its permeability to potentially neurotoxic or proinflammatory molecules and cytokines [4]. In addition, constitutive depletion of the gut microbiota in germ-free mice markedly compromises the maturation of microglia, the brain resident macrophages, leading to impaired innate immune responses [5]. However, orally administered SCFA are sufficient to restore microglia morphological alteration, supporting an important role of these bacterial metabolites in modulating the biology of brain resident macrophages [5]. This last point is of interest in the context of brain/gut connection, as microglia are very unique among tissue-resident macrophages. Adult microglia differ from their peripheral counterparts by a particular origin from embryonic yolk sac progenitors, a ramified morphology with highly motile processes monitoring any change in their environment, and an actively repressed surveying phenotype (CD45^low^) [6,7,8]. Consistent with their diverse roles, microglial activation, while necessary for central nervous system (CNS) protection, have also been linked to the initiation or progression of several developmental and neurodegenerative diseases [9]. Besides their role in neuroinflammation, bacterial metabolites directly or indirectly impact neuronal function, connectivity, and survival. SCFA plays a prominent role by modulating the production of neuroactive hormones or neurotrophic factors [2,10,11]. Bacteria can also directly produce neurotransmitters such as GABA and 5-hydroxytryptophan (5-HT) [12] or can indirectly stimulate 5-HT production in the gut through the production of metabolites like SCFA and secondary bile acids [13]. Microbiota composition and metabolites are altered in different brain disorders such as neurodevelopmental, neurodegenerative, and psychiatric diseases, often associated with gastrointestinal (GI) comorbidities [14]. In many cases, microbiota transfer experiments in gnotobiotic animals have shown that the altered microbiota can transfer part of the intestinal or central symptomatology. This has led to consider microbiota modulating strategies as a promising way to improve the quality of life or even the severity of symptoms for patients suffering from brain disorders [15].

In this context, few recent studies have focused on the role of gut microbiota in brain tumor development. However, the microbiota is involved in the development and treatment efficacy of different digestive and extraintestinal cancers [16,17]. More specifically, bacterial metabolites such as bile acids [18,19], inosin [20], adenosine derivatives [21], and SCFA [22] are key beneficial or deleterious mediators in cancer development or treatment. Gliomas are the main primary brain cancers in adults and children and arise from the malignant transformation of glial cells (astrocytes, oligodendrocytes) in the Central Nervous System (CNS). Glioma stroma is also infiltrated by resident microglia and newly recruited bone marrow derived-macrophages which represent up to 30% of the tumor mass [23]. In contrast to resident microglia, glioma-associated microglia are rapidly activated, exhibiting high levels of Cluster of Differentiation 45 (CD45), making them indistinguishable from infiltrated macrophages [24]. Glioma-associated microglia/macrophages (GAM) tend to be pro-tumorigenic, and their accumulation negatively correlates with patient survival [25]. This cancer is generally associated with a dismal prognosis and few effective therapeutic options. A better understanding of glioma physiopathology may thus open a new perspective for this disease’s clinical management. Recent studies showed that glioma is associated with a shift in gut microbiota composition [26] and fecal bacterial metabolites, including SCFA and neurotransmitters in mice and patients [27]. Similarly, a significant shift in the circulating extracellular vesicle microbiome has been observed between healthy and brain tumor patients [28].

In the present study, we used a mouse model to determine the consequence of glioma development on GI functions and host/microbiota interaction in vivo. We show that late-stage glioma is associated with significant changes in GI motricity and a shift in the composition of cecal metabolites, which speed up mice death upon oral transfer.

## 2. Results

### 2.1. Late-Stage Glioma in Mice Is Associated with Changes in Gut Motricity and Expression Level of Genes Involved in Epithelial Antimicrobial Defense in the Jejunum and Ileum

**Figure 1:**
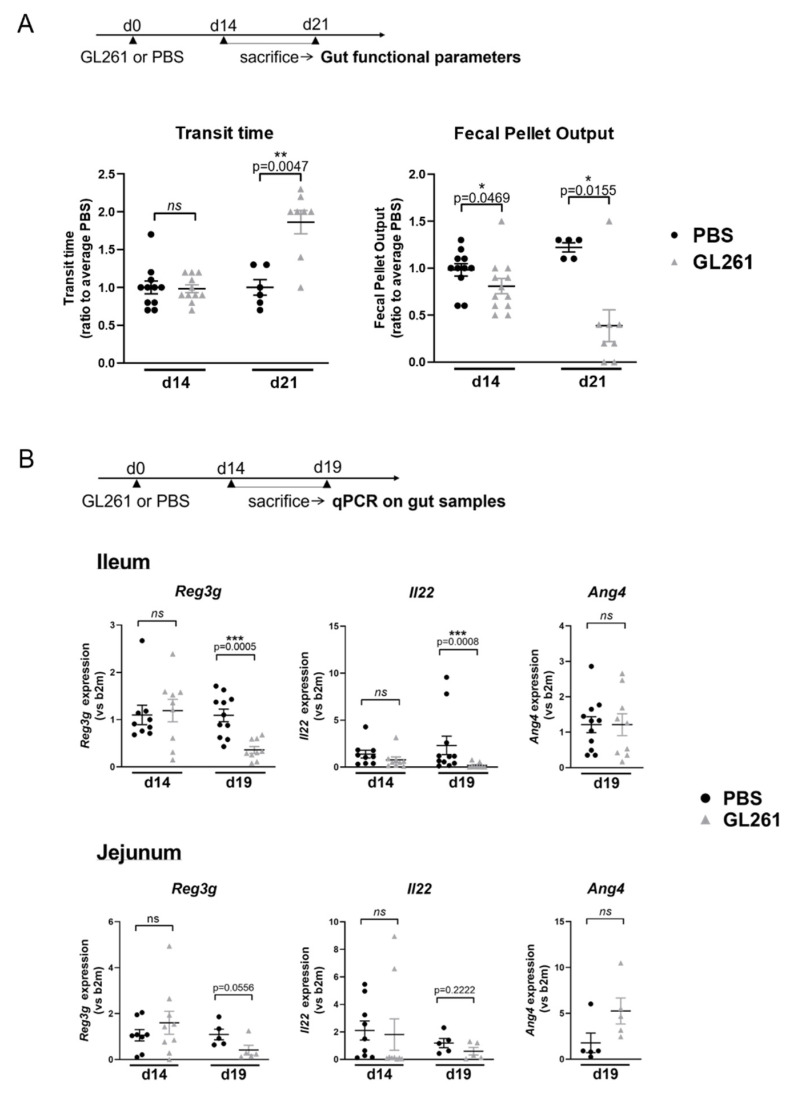
Changes in gastrointestinal motricity and expression level of genes related to small intestinal immunity in mice with late-stage glioma (**A**) Total transit time and fecal pellet output in mice injected with GL261 or PBS, 14 days (d14) and 21 days (d21) after the surgery. Transit time represents the latency before rectal expulsion of orally administered carmine red. Fecal pellet output is the number of stools expulsed in 2 h. Parameters are expressed as percentages of the average value of PBS-treated mice. All values represent means ± SEM (PBS: *n* = 5–6; GL261: *n* = 5–8 for d21 and PBS: *n* = 11; GL261: *n* = 11–12 for d14). Statistical analyses were performed with the Mann–Whitney *U*-test, * 0.01 ≤ *p* < 0.05, ** 0.001 ≤ *p* < 0.01 (**B**) Expression level of genes involved in epithelial antimicrobial defense (*Reg3γ*, *Il22*, *Ang4*) in the ileum and jejunum of mice 14 days (d14) and 19 days (d19) after the surgery. Expression levels are expressed in reference to the *b2m* housekeeping gene and have been normalized to the average gene expression in PBS-injected mice. All values represent means ± SEM (PBS: *n* = 11; GL261: *n* = 9 for the ileum and PBS: *n* = 4–5; GL261: *n* = 5 for jejunum for d19 and PBS: *n* = 8–9; GL261: *n* = 9 for d14). Statistical analyses were performed with the Mann–Whitney *U*-test, *** 0.0001 ≤ *p* < 0.001, ns: non-significant.

We used here a syngeneic model of glioma in mice, based on the orthotopic inoculation of the GL261 cell line in immunocompetent C57BL6 strain. Following the injection of 5000 cells at day 0, mice were monitored daily and were sacrificed if any sign of pain was detected or if the mice lost more than 10% of their weight, in accordance with ethical guidelines. In these experiments, all mice died within 30 days following tumor implantation. In order to study gastrointestinal comorbidities associated with glioma progression in this model, we monitored different gut functional parameters, 14 and 21 days following tumor implantation. On day 21, total transit time and fecal pellet output (FPO) were significantly increased and decreased, respectively, in glioma-bearing mice, compared to sham-operated mice injected with phosphate buffered saline (PBS) (Figure 1A). In contrast, the transit time was not altered at day 14, although a slight but significant decrease in FPO was already detectable at this earlier time point (Figure 1A). Gut paracellular and transcellular permeabilities were also monitored in vivo by quantifying the blood level of orally administered fluorescent sulfonic acid (FSA) and horseradish peroxidase (HRP), respectively. Gut permeability was not altered in glioma-bearing mice, neither at day 14 nor at day 21 following GL261 inoculation (Figure S1A). Similarly, gut permeability across isolated GI regions (jejunum, ileum, proximal colon), studied in Ussing chambers, was unchanged in glioma-bearing mice (Figure S1B). Finally, stool hydration was unaltered in glioma-bearing mice, neither at day 14 nor at day 21 (Figure S1A).

In parallel with these functional studies, we analyzed by RT-qPCR the expression level of genes involved in (i) epithelial functions, including antimicrobial defense (*Reg3g*, *Il22*, *Ang4*), differentiation (*Lgr5*, *Math1*, *Hes1*, *Spdef*, *Alpi*), and permeability (*Cldn1*, *Occldn*, *Zo1*), and (ii) in mucosal inflammation (*Il1b*, *Tnfa*, *Il6*) and immune balance (*Rorc*, *Foxp3*, *Il17*, *Ifng*, *Tgfb*, *Ccl20*) in different regions of the GI tract (jejunum, ileum, and proximal colon) 14 or 19 days after tumor implantation. A significantly reduced mRNA expression of the antimicrobial peptide (AMP) *Reg3g* was only found at day 19 in the jejunum and ileum of GL261-bearing mice (*p* = 0.0556 and *p* = 0.0005, respectively) (Figure 1B), although no significant change was observed in the proximal colon (Figure S2). On day 19, we also observed a significant decrease in *Il22* mRNA expression in the ileum (*p* = 0.0008), a cytokine known to induce the expression of *Reg3g* in epithelial cells (Figure 1B). These modifications were not associated with a reduced expression of *Ang4*, another AMP gene, nor with any change in the expression level of genes encoding transcription factors driving epithelial cells differentiation, including those driving secretory lineage differentiation (*Math1*, *Spdef*) (Figure 1B and Figure S2). In accordance with our functional studies on permeability, no difference in the levels of mRNA encoding tight junctions proteins (*Cldn1*, *Zo1*, *Ocln*) and inflammatory cytokines (*Il1b*, *Tnfa*, *Il6*) was observed in the jejunum, ileum, or proximal colon between glioma-bearing and healthy mice (Figure S2). Likewise, we did not find any change in the expression of genes involved in mucosal immune balance in the ileum or proximal colon either (Figure S2).

### 2.2. Late-Stage Glioma Is Associated with a Shift in Bacterial Metabolites in the Cecum That Contributes to Speed up Mice Death upon Transfer

**Figure 2:**
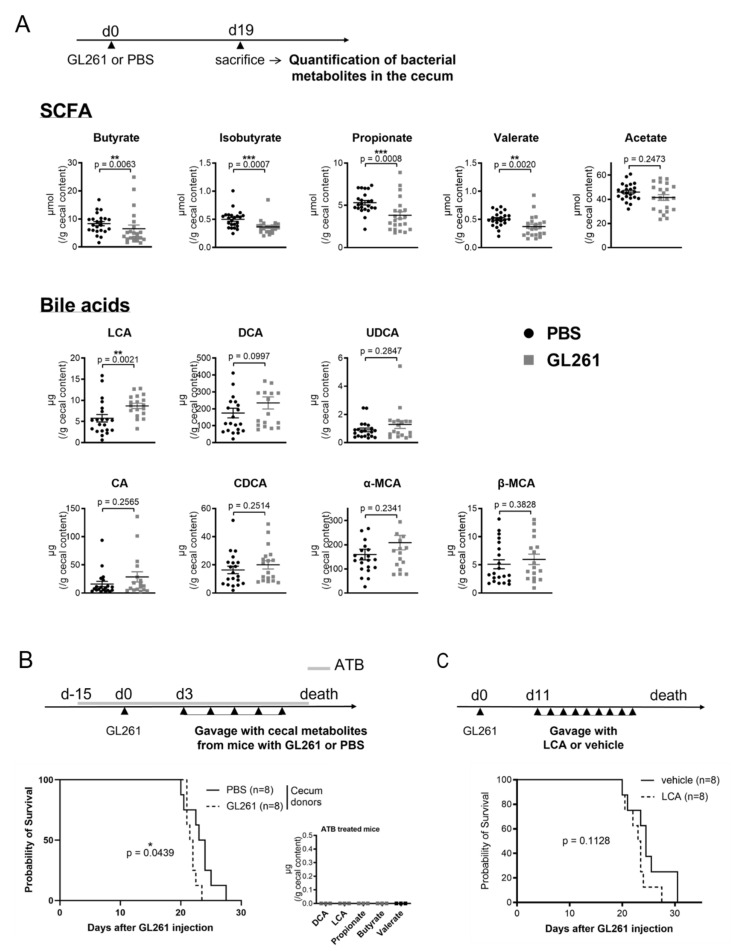
Late-stage glioma is associated with a shift in bacterial metabolites in the cecum, which speed up mice death upon transfer (**A**) Quantification of short-chain fatty acids (SCFA) (PBS *n* = 23; GL261 *n* = 22) and bile acids (PBS *n* = 20–21; GL261 *n* = 17–18) in the cecum of mice injected with GL261 or PBS, 19 days after the surgery. UDCA: ursodeoxycholic acid; DCA: deoxycholic acid; LCA: lithocholic acid; CA: cholic acid; CDCA: chenodeoxycholic acid; MCA: muricholic acid. Statistical analyses were performed with the Mann–Whitney *U*-test, ** 0.001 ≤ *p* < 0.01, *** 0.0001 ≤ *p* < 0.001. (**B**) Kaplan–Meier survival curve of mice, treated with antibiotics (ATB) fifteen days before GL261 inoculation and fed with the cecal content of tumor-bearing (GL261) or healthy (PBS) mice every other day from day 3. The experiment was performed with 8 mice per group and the survival curves were compared using a long-rank test. The level of secondary bile acids (LCA and DCA) and short-chain fatty acids (propionate, butyrate, and acetate) has been quantified in the cecum of GL261-bearing mice treated with broad-spectrum antibiotics (ATB) for 34 days. * 0.01 ≤ *p* < 0.05 (**C**) Survival of GL261-bearing mice fed with LCA or vehicle (sunflower oil) every day from day 11. The experiment was performed with 8 mice per group and statistics were done using a log-rank test.

Our gene expression analysis showed that the antimicrobial peptide *Reg3g* mRNA is downregulated in the jejunum and ileum of mice with late-stage glioma. Knowing that REG3γ is involved in shaping the gut microbiota composition [29], we speculated that late-stage glioma could be associated with a shift in bacterial metabolites, which are important mediators allowing the microbiota to modulate host physiology, including the brain. We thus quantified the levels of targeted bacteria-derived metabolites in the cecal content of glioma-bearing and healthy mice by mass spectrometry. We focused our analysis on five short-chain fatty acids (SCFA) and seven unconjugated bile acids, as these are produced by bacteria in the gut. These metabolites were chosen based on their involvement in brain/gut communication and cancer development or treatment [1,18,19,22,30]. The concentration of four SCFA, namely butyrate, isobutyrate, propionate, and valerate, was reduced in the cecum of glioma-bearing mice, compared to PBS-injected mice (Figure 2A). On the other hand, the level of the secondary bile acid lithocholic acid (LCA) was significantly increased in the cecum of GL261-bearing mice, whereas the level of all other bile acids was unchanged between glioma-bearing and healthy mice (Figure 2A).

Based on this finding, we wonder if such alteration in the concentration of cecal bacterial metabolites could impact the kinetic of mice death. To address this question, we performed cecal content transfer experiments. The cecum of tumor-bearing (GL261) and healthy (PBS) mice were harvested at sacrifice (day 19) and cecal metabolites were obtained following homogenization and sterile filtration. Cecal content was administered to GL261-bearing mice every other day by oral gavage, starting 3 days after the surgery and until the end of the experiment (Figure 2B). Recipient mice were fed with broad-spectrum antibiotics all along the experiment to deplete endogenous bacterial metabolites. The antibiotic cocktail contains five different antibiotics (amphotericin B, neomycin, vancomycin, ampicillin, and metronidazole) and is known to drastically reduce the microbial load in the gut [31]. Bacterial metabolites such as the secondary bile acids LCA and DCA and the SCFA propionate, butyrate, and valerate were indeed undetectable in the cecum of antibiotic-treated GL261-bearing mice (Figure 2B). Antibiotic-treated mice receiving cecal metabolites from GL261-bearing mice died slightly but significantly earlier than those receiving cecal metabolites from healthy mice (*p* = 0.0439) (Figure 2B). To study the involvement of one specific bacterial metabolite in this effect, we fed mice with LCA, a secondary bile acid that was enriched in the cecum of mice with late-stage glioma. Although the results did not reach significance, mice fed with LCA tend to die earlier than vehicle-treated mice (*p* = 0.1128) (Figure 2C).

### 2.3. Oral Antibiotherapy Delay Mice Death and Is Associated with a Shift in the Proportion of Immune Myeloid Cells in the Brain Tumor Environment

**Figure 3:**
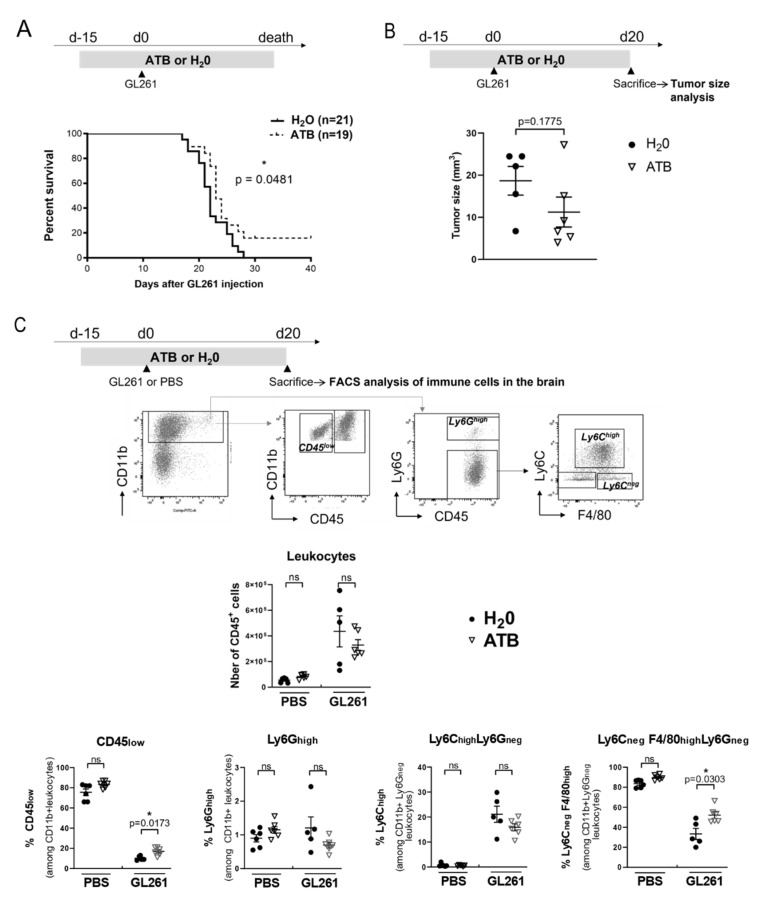
Antibiotic treatment delays mice death and changes the proportion of different myeloid cells in the brain (**A**) Survival curves of mice injected with GL261 and treated daily with antibiotics (ATB) (*n* = 19) or H_2_O (*n* = 21) by oral gavage 15 days before the surgery and until sacrifice. Log-rank test compared with control: see the indicated *p*-value (**B**) Tumor size of mice injected with GL261 (*n* = 6) or PBS (*n* = 5), 20 days after the surgery. (**C**) Absolute number and proportion of different immune cells (CD45^+^) in the brain of tumor-bearing (GL261; H_2_O *n* = 5; ATB *n* = 6) and healthy mice (PBS; H_2_O *n* = 6; ATB *n* = 6), by flow cytometry (FACS). A representative gating strategy is depicted. Mann–Whitney test compared ATB and H_2_O treated mice, * *p* < 0.05.

To gain further insight into the mechanisms underlying the deleterious effect of the gut microbiota on mice death, we treated glioma-bearing mice with broad-spectrum antibiotics (ATB) to drastically reduce the microbial load in the gut. This ATB cocktail did not induce any direct cytotoxicity on GL261 cells in vitro (Figure S3). Mice were fed daily with ATB or water (H_2_O) 15 days before tumor inoculation and until the end of the experiment. ATB treatment slightly but significantly delayed the death of mice (Figure 3A). A decrease in FPO 14 days after the surgery was still observed in ATB-treated mice, suggesting that these early glioma-associated gut functional alterations may partially be independent of the microbiota (Figure S3). We then repeated this experiment by sacrificing mice at day 20 to analyze the size of the tumors and the immune environment of the brain by flow cytometry. Although we did not find a significant difference in tumor size between ATB and H_2_O treated mice (Figure 3B), we found that ATB treatment significantly changed the frequency of myeloid cells in the brain tumor environment. Hence, ATB treatment was associated with an increased proportion of resident microglia (CD45^low^ CD11b^+^ cells) and of glioma-associated microglia/macrophages (GAM, CD45^high^ CD11b^+^ F4/80^+^) in the brain, specifically in GL261-bearing mice (Figure 3C) (*p* = 0.0173 and *p* = 0.0303, respectively). In addition, while being absent in the healthy brain, peripheral Ly6C^high^ monocytes were increased in the CNS of glioma-bearing mice treated or not with ATB.

## 3. Discussion

In this study, we showed that late-stage glioma in mice is associated with alteration in gut functions and with a shift in the concentration of bacterial metabolites in the cecum. This is in accordance with recent studies showing that glioma is associated with gut dysbiosis and with the altered fecal level of metabolites, including SCFA, both in mice and humans [26]. Our work further shows that such cancer-associated alteration in bacterial metabolites is deleterious for end-stage glioma lethality. We identified LCA as a potential candidate metabolite involved in glioma lethality. This metabolite has already been involved in non-glioma tumor progression, playing a dual role in promoting or inhibiting tumor growth, depending on the type of cancer. LCA is one of the most hydrophobic bile acids and is thus considered one of the most toxic due to its ability to disrupt the cell membrane. Like all other bile acids, it is also a signaling molecule, which can interact with the membrane-bound receptor TGR5 and with the nuclear receptors FXR (farsenoid X receptor), VDR (vitamin D receptor), and PXR (pregnane X receptor). In vitro studies have shown that LCA can kill breast, prostate, and neuroblastoma cancer cells [32,33,34,35]. On the opposite, reports demonstrating the potent carcinogenic property of LCA in colorectal cancer (CRC) were published more than 40 years ago. Whereas LCA can promote the development of chemically induced CRC in animal models [36], an elevated LCA/DCA fecal ratio is a known CRC risk factor in humans [37,38]. The pro-tumoral role of LCA has also been shown in liver and pancreatic cancers using animal models [18,39]. On top of its direct DNA damaging effect, LCA has been shown to modulate cell survival and also promote cancer stem cells phenotype [40,41,42]. Besides its role in cancer progression, LCA has also been involved in other CNS-related pathologies. Patients who have Alzheimer’s disease (AD) have increased blood levels of LCA, which is thus considered a potential noninvasive marker for the diagnosis of the disease [43]. The authors showed that LCA blood level could increase by 3.2-fold when healthy individuals convert to AD patients, along an 8–9 year follow-up period. Similarly, LCA plasma level is increased in APP/PS1 mice, a transgenic model of AD [44]. In glioma-bearing mice, we also observed a decrease in short-chain fatty acids such as isobutyrate, butyrate, or propionate in the cecal content from GL261-bearing mice. Knowing the role of SCFA in brain health [2], their decreased level may also be involved in the deleterious effect of cecal metabolites observed in this model. Further studies are needed to unravel the role of SCFA and LCA in glioma-associated mortality. Furthermore, performing untargeted metabolomics will be useful to unravel the role of other bacterial metabolites in glioma progression. Similarly, combined metagenomic and metabolomic analyses are required to fully understand the link between microbiota phylogenetic composition and bioactive bacterial metabolites. Interestingly, bacteria belonging to the Bacteroidetes Phylum, particularly the S24–7 family, with potential propionate-producing properties, were found to be decreased in the microbiome of brain tumor patients in two independent studies [28,45]. Similarly, SCFA-producing genera such as Eubacterium, Dialister, Blautia, and Bifidobacterium were found to be less abundant in the circulating extracellular vesicle microbiome of brain tumor patients [28]. In particular, the genus Dialister and the species *Eubacterium rectale* were also significantly decreased in the brain of these patients [28]. Of note, Dialister and *E. rectale* were recently found to be decreased in the fecal metagenomic signature shared among 5 to 8 different extra-intestinal cancers [46].

Late-stage glioma was characterized by altered gut functional parameters, notably by an increased total transit time and decreased fecal pellet output. Gut microbiota and, in particular, bacterial metabolites, including SCFA and bile acids, are known to impact gut transit [13,47]. Hence SCFA, and, in particular, butyrate, can have both positive and negative effects on colonic motility, depending on its concentration [13]. Similarly, bile acids are traditionally considered as potent natural laxatives, thus having a positive effect on colonic motility while reducing the small intestinal motility [48]. Although LCA increases colonic peristalsis in preclinical models [49], it was found to be increased in the stool of constipated irritable bowel syndrome patients [50]. Interestingly, serotonin, a regulator of gut transit, was altered in the stool of glioma-bearing mice in another study, suggesting a potential link between dysbiosis and gut transit in this model [27]. Although we did not study deeply the causal relationship between gut functional alteration and dysbiosis, we found that the early decrease in Fecal Pellet Output (FPO) may be partially independent of the microbial alteration as it occurred in microbiota-depleted antibiotic-treated mice (Figure S3). Further studies are required to fully address the cause of gut functional alteration in glioma. The consequence of the presence of a tumor in the brain for brain/gut communication as well as the role of decreased food intake at the late stage of the disease may also be considered in this regard. Based on the early decrease in colonic FPO, one appealing hypothesis may be that gut transit alteration could be the first step, leading to subsequent microbiota alteration in this model [51].

Our study shows that antibiotic treatment aimed at depleting the microbiota delays mice death and changes the immune environment of the brain. In particular, we found that antibiotic treatment increased the proportion of both resident microglia (CD45^low^ F4/80^+^) and glioma-associated microglia/macrophages (GAM) (CD45^high^ F4/80^+^). Although GAM have been shown to play a deleterious role in glioma progression [52], the role of resident microglia has not been fully elucidated yet, due to the lack of markers to differentiate the two cell population. Resident microglia and macrophages, ontogenetically distinct, can have very different roles, often opposed, in neuroinflammation [53], and their study in the context of brain tumors requires further investigation. Conversely, dysbiosis inducing antibiotherapy has recently been shown to have a deleterious effect on glioma progression by inhibiting natural killer cells mediated cancer immunosurveillance, while no change in the microglia population was observed in this study [54]. This apparent discrepancy with our results may partly rely on the fact that we used a different antibiotic cocktail with a more drastic effect on the microbiota. Hence the microbiota may have both positive and negative effects on brain tumor development, and different bacteria or metabolites may have the opposite effect on the progression of the pathology. Further studies are thus needed to unravel the role of bacteria or bacterial metabolites on the glioma immune environment and the consequence for disease-associated mortality. The use of microbiota transfer experiments in gnotobiotic animals may be relevant to exclude any direct effect of the ATB on host physiology that may be independent of their microbiota-depleting effect.

The terminal stage of cancer is often associated with cachexia, a metabolic disorder characterized by a refractory weight loss that impairs treatment efficiency and precipitates a patient’s death. Cachexia hallmarks include systemic inflammatory cytokines production, like IL-6, weight loss, and gut barrier dysfunction [55]. In our study, we did observe a drastic and irreversible weight loss at late-stage glioma. However, this was not associated with alteration in gut permeability and inflammation, in contrast to what is observed in the non-glioma model of cancer cachexia in mice [56]. *Reg3g* downregulation in the absence of drastic inflammation may thus be a characteristic of a glioma-associated pre-cachexic state. Dysbiosis is also a hallmark of cachexia [57] and microbiota modulating strategies, including probiotics and SCFA generating prebiotics, are promising strategies to attenuate cachexia in cancer patients [58]. Our study gives a rationale to test these strategies in glioma patients.

## 4. Materials and Methods

### 4.1. Animals

C57BL/6 male and female mice were purchased from Envigo (Gannat, France) and used between 8- and 10-weeks-old in Angers hospitalo-university-specific pathogen-free facility (SCAHU).

Animal protocols were approved by the institutional ethics committee of the Region Pays de la Loire and authorized by the French ministry of research (APAFIS 7639-201611181450984).

### 4.2. GL261 Tumor Cells Brain Injection

The murine glioma cell line GL261 (C57BL/6 strain, H2-b) was kindly provided by Dr. Paul Walker (Geneva, Switzerland). Cells were cultured in DMEM containing 4.5 g/L of glucose, supplemented with 10% heated-inactivated fetal calf serum, 2 mM L-glutamine, 0.1 mM non-essential amino acid, 10 mM Hepes, 1 mM sodium pyruvate, 100 U/mL penicillin, and 100 µg/mL streptomycin (all from Lonza, Verviers, Belgium).

Cells were trypsinized and washed twice in PBS before brain implantation into 8 to 10-week-old syngeneic mice, according to the previously described protocol [59]. Briefly, mice were anesthetized by an intraperitoneal injection of a mixture of ketamine (10 μg/g) and xylazine (1 μg/g) and were held in a stereotaxic frame (Stoelting, Dublin, Ireland). 0.5 or 1 × 10^4^ GL261 glioma cells in 2 μL sterile PBS or PBS alone were injected (0.5 μL/min) in the caudate-putamen of the right hemisphere, with the following coordinates: anterior −0.5 mm, lateral 2 mm, depth −2.5 mm from the bregma. The needle was held in position for a further minute and withdrawn slowly to avoid backfilling of the solution. Animals were observed daily, reduced mobility and significant weight loss (10%) were considered as the endpoint for survival curves.

### 4.3. Antibiotic and Lithocholic Acid (LCA) Treatment

Sterile filtered ampicillin (Sigma A9518) was put in the drinking water at a concentration of 1 g/L and changed once a week. A solution containing metronidazole 10 mg/mL (Sigma M3761), vancomycin 5 mg/mL (Sigma V2002), neomycin 10 mg/mL (Sigma N1876) and amphotericin B 0.1 mg/mL (Sigma A9528) was administered daily by oral gavage (10 mL/kg body weight). The treatment started two weeks before tumoral cells inoculation and lasted until the end of the experiment. LCA (Sigma L6250) was dissolved in sunflower oil and administered daily to non-antibiotic treated mice by oral gavage (0.25 mg/g body weight), from day 11 following GL261 cells injection and until the end of the experiment.

### 4.4. Cecal Metabolites Transfer Experiment

The cecum from mice injected with GL261 or PBS were taken 19 days after the stereotaxic surgery. The cecum content was weighed, homogenized in ice-cold HBSS (10 mL HBSS/g cecum content), and centrifuged at 3000× *g* for 15 min at 4 °C. The supernatant was then passed through a 70 μm cell strainer and sterile filtered. Cecal contents from 5–10 mice were pooled, aliquoted, and kept at −80 °C. Recipient mice were treated with antibiotics (see the previous section) during all the experiments and were fed every other day with 200 μL of pooled cecal content, starting 3 days after the stereotaxic surgery and until the end of the experiment.

### 4.5. In Vivo and Ex Vivo Intestinal Parameters Monitoring

Total transit time, fecal pellet output, and in vivo and ex vivo permeability were measured as previously described [60,61,62]. Mice were fed by oral gavage with a solution (120 µL/animal) containing Fluorescein-sulfonic acid (FSA) 10 mg/mL (LifeTechnologies F1130), Horseradish peroxidase (HRP) 10 mg/mL (Sigma P8250-200KU) and Carmine red 60 mg/mL (CHU of Nantes) dissolved in 0.5% carboxymethylcellulose (Sigma C4888). Total transit time was obtained by monitoring the time between the gavage and the excretion of the first red stool. Fecal pellet output was studied by counting the number of stools expulsed by each mouse during a 2 h follow-up period. Stools were weight and their water content was studied by weighing them following a 48 h incubation in a dry incubator at 37 °C.

For in vivo gut permeability study, FSA and HRP were used as markers of paracellular and transcellular permeability, respectively. Concentrations of FSA and HRP in the blood plasma were evaluated 2 h following the initial gavage. FSA concentration was assessed by measuring the fluorescence (λex. 488 nm, λem. 520 nm) of plasma using a fluorimeter microplate reader (Varioskan, Thermo, Courtaboeuf, France). HRP quantification in plasma was performed using an enzymatic assay based on tetramethylbenzidine (TMB) substrate (Becton-Dickinson, 555214) and optical density was measured thanks to a spectrophotometer microplate reader at 450 nm (Varioskan, Thermo, Courtaboeuf, France). In both cases, a standard curve of FSA and HRP was done using stock solutions.

Ex vivo intestinal permeability was assessed using Ussing chambers. Freshly removed segments of jejunum, ileum, and proximal colon were opened along the mesenteric border and mounted in dedicated Ussing chambers (Physiological instruments, San Diego, CA, USA, 0.031 cm^2^ surface). Apical and basolateral sides of tissue were incubated in 2 mL of F12 supplemented Dulbecco’s Modified Eagle medium (Life Technologies 31330038) containing 0.1% fetal bovine serum (Biowest), 2 mM Glutamine (Life Technologies 25030024), and 45 g/L of NaHCO_3_ (Sigma S5761). The medium was continuously oxygenated with a 95% O2/5% CO_2_ gas flow and maintained at 37 °C. Once equilibrium was reached, 30 min after tissues had been mounted, 200 µL of the apical medium was replaced with 200 µL of medium containing FSA (1 mg/mL) and HRP (3.75 mg/mL). To assess paracellular permeability, the fluorescence levels of 150 μL aliquots taken at the basolateral side were measured at 30 min intervals over a period of 180 min using a fluorimeter microplate reader. To assess permeability to HRP, 30 µL aliquots from the basolateral side were recovered at 1 h intervals for 3 h and HRP activity was determined as previously described. Paracellular permeability to FSA and transcellular permeability to HRP were determined by calculating the slope of the changes, respectively, in fluorescence intensity and DO intensity over time using a linear regression fit model (GraphPad Software Company, La Jolla, CA, USA).

### 4.6. Gene Expression Analysis in Mouse Tissues

For gene expression analysis, RNA was extracted from tissues using the Nucleospin RNA protein kit (Macherey-Nagel 740933). Initial tissue disruption and homogenization were performed in the lysis buffer using Precellys homogenizer (Ozyme) for 2 × 15 s with “back-and-forth movement” at a frequency of 6500/min. Following RNA extraction, reverse transcription was performed using the Superscript II reverse transcriptase (Fisher Scientific) and random hexamers.

Quantitative RT-PCR experiments were performed by using Power SYBR Green mix (Applied). Primers were designed and validated by the PACEM platform of the University of Angers. Relative gene expression was calculated with the standard delta Ct method using *β2m* as the reference housekeeping gene. Data are normalized to the control condition, which is the average of gene expression in healthy (PBS) mice. Specific expression levels are obtained according to the following formula: 2^((Ct_b2m_-Ct_gene_)-average _PBS mice_ (Ct_b2m_-Ct_gene_)).

### 4.7. Quantification of Cecal Metabolites

Fecal bile acids, including LCA, ursodeoxycholic acid (UDCA), deoxycholic acid (DCA), cholic acid (CA), chenodeoxycholic acid (CDCA), and muricholic acid (MCA), were quantified from ~50 mg of fecal samples by liquid chromatography-tandem mass spectrometry (LC-MS/MS) as described previously [63]. Short-chain fatty acids (SCFA), including acetate, propionate, butyrate, isobutyrate, and valerate, were quantified from ~20 mg of fecal samples by gas chromatography-mass spectrometry as described previously [64].

### 4.8. Tumor Size Analysis

Brains were removed at sacrifice and were fixed for 24 h in 4% paraformaldehyde. Serial slices of 400 µm were cut using a vibratome. Tumor areas were analyzed using ImageJ software and tumor volume was calculated according to the formula: volume = t.A, where t is the thickness of the slices (400 µm) and A is the sum of all the tumor areas.

### 4.9. Flow Cytometry Analysis of Immune Cells in the Brain

According to ethical guidelines, brain cells from mice sacrificed using cervical dislocation were isolated as previously described [65]. Briefly, the brain was isolated from mice and cerebellum and meninges were removed, dilacered, and digested using 0.25% trypsin (Sigma) and 0.1 mg/mL DNAse 1 (Sigma 11284932001) in HAM-F12 medium (Lonza BE12-615F) for 10 min at 37 °C under 5% CO_2_. The cell suspension was then passed through a 70 µm cell strainer and separated using a percoll gradient with 3 phases: 70%–37%–30%. Cells at the interphase between percoll 30% and percoll 37% were collected, washed, and numerated.

After incubation with anti-CD16/CD32 mAb (clone 2.4G2, rat IgG2b) to saturate Fc receptors, staining for flow cytometry was done using the following antibodies recognizing mouse antigens: CD45-AF647 (clone 30F11, Biolegend); CD11b-APC (clone M1/70, Biolegend); Ly6C-V450 (clone AL-21, BD Biosciences); Ly6G-FITC (clone 1A8, BD Biosciences), F4/80-PE (clone BM8, Molecular probes) or isotype-matched controls. Dead cells were excluded using the 7AAD viability marker (Invitrogen). Data were analyzed using the FlowJo software (version 10) (Tree Star, Ashland, OR, USA).

### 4.10. Statistics

Data are expressed as mean +/− SEM. Mann–Whitney test was performed. *p*-value < 0.05 was considered significant. Survival curve comparison was made using the Log-rank test (Mantel-Cox). Statistics were done using GraphPad Software (version 8) Outliers were identified with the ROUT method.

## 5. Conclusions

Late-stage glioma in mice is associated with a deleterious alteration of the cecal content, characterized by a shift in the concentration of different bacterial metabolites like SCFA and secondary bile acids. Future studies may fully characterize the fecal, circulating, and brain metabolomic alterations associated with late-stage glioma, both in mice and patients. This work gives a rationale to consider microbiota-modulating strategies, including diet intervention, for the clinical management of glioma patients.

## Data Availability

Data supporting the figures of this manuscript have been deposited in the Biostudies database of the European Bioinformatics Institute (EMBL-EBI) under the accession number S-BSST796 (https://www.ebi.ac.uk/biostudies/studies/S-BSST796, accessed on 3 February 2022).

## Supplementary Materials

The following supporting information can be downloaded at: https://www.mdpi.com/2218-1989/12/4/290/s1. Figure S1. Gut permeability in mice with late stage glioma; Figure S2. Expression of genes involved in epithelial permeability (*Occldn*, *Zo1*, *Cldn1*), antimicrobial defense (*Reg3g*, *Il22*, *Ang4*) and differentiation (*Lgr5*, *Math1*, *Hes1*, *Spdef*, *Alpi*), and in mucosal inflammation (*Il1b*, *Tnfa*, *Il6*) and immune balance (*Rorc*, *Foxp3*, *Il17*, *Ifng*, *Tgfb*, *Ccl20*), in different GI regions in mice injected with GL261 or PBS and sacrified at day 19; Figure S3. Impact of the antibiotic cocktail on GL261 viability in vitro and on gut functional parameters in vivo.
