# Supplementary material for: Late-Stage Glioma Is Associated with Deleterious Alteration of Gut Bacterial Metabolites in Mice
Source: Metabolites. 2022 Mar 25;12(4):290. doi: 10.3390/metabo12040290 (PMC9028041; doi:10.3390/metabo12040290)
Supplement: Supplementary file 1 [file metabolites-12-00290-s001.zip › metabolites-1605627-supplementary.pdf]

Supplemental Figure 1

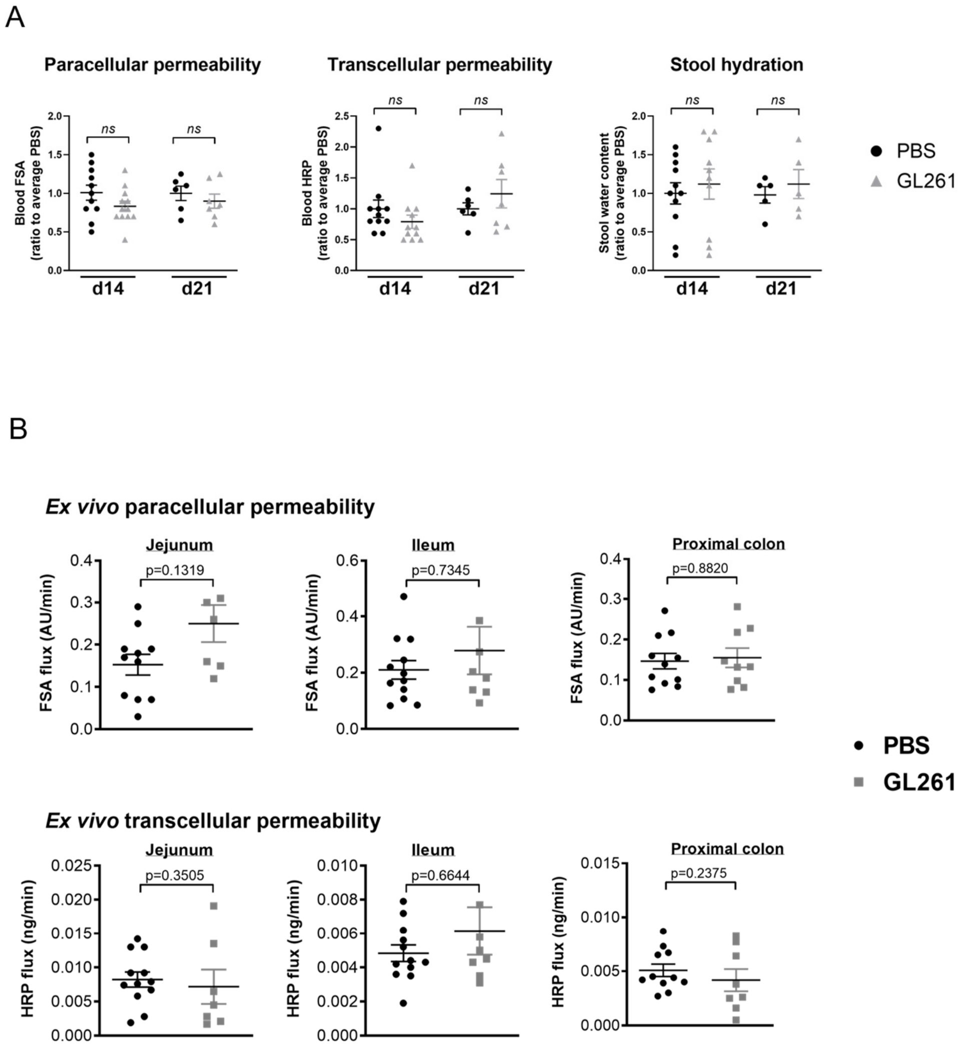

Sup Figure S1. Gut permeability in mice with late stage glioma. (A) Gut permeability and stool hydration in mice injected with GL261 or PBS, 14 days (d14) and 21 days (d21) after the surgery. Paracellular and transcellular permeabilities were measured by quantifying the blood level of sulfonic acid (FSA) and horse raddish peroxidase (HRP) respectively 2 hours after their oral administration, and are expressed as percentages of the average value of PBS treated mice. All values represent means $\pm$ SEM. Statistical analyses were performed with the Mann-whitney U-test, * $p<0.05$. **(B)** Paracellular and transcellular permeability across different regions of the GI tract (jejunum, ileum and proximal colon), 19 days after GL261 or PBS injection. Transcellular and paracellular permeabilities were monitored by quantifying the flow of HRP and FSA respectively across different GI regions *ex vivo* in Ussing chambers. All values represent means $\pm$ SEM (PBS : n= 11-12 ; GL261 : n=7-9). Statistical analyses were performed with the Mann-whitney U-test, * $p<0.05$.

Supplemental Figure 2

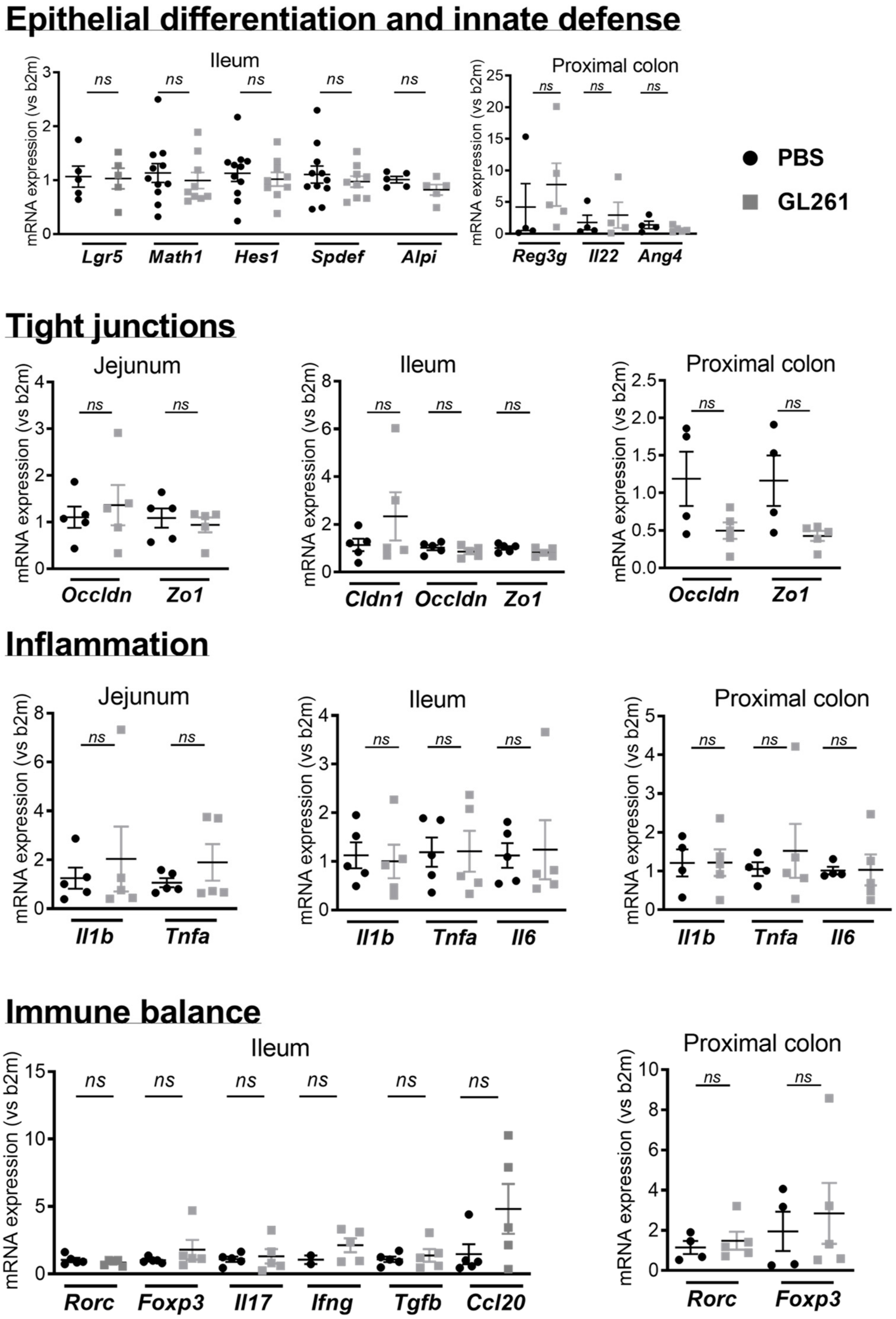

Sup Figure S2. Expression of genes involved in epithelial permeability (*Occldn, Zo1, Cldn1*), antimicrobial defense (*Reg3g, Il22, Ang4*) and differentiation (*Lgr5, Math1, Hes1, Spdef, Alpi*), and in mucosal inflammation (*Il1b, Tnfa, Il6*) and immune balance (*Rorc, Foxp3, Il17, Ifng, Tgfb, Ccl20*), in different GI regions in mice injected with GL261 or PBS and sacrified at day 19. Expression level is expressed in reference to *b2m* housekeeping gene and has been normalized to the average expression in PBS injected mice. All values represente means ± SEM (PBS : n= 4-11 ; GL261: n= 5-9).

Supplemental Figure 3

A

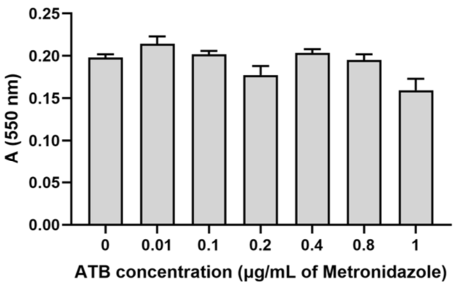

B

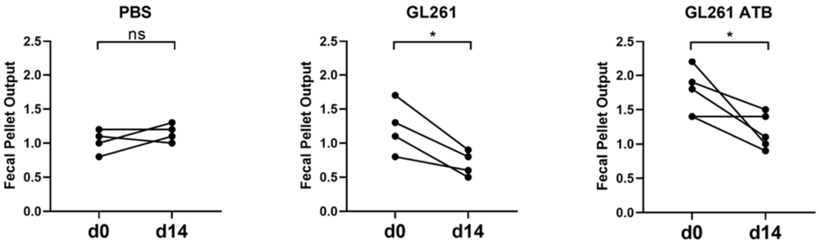

Sup Figure S3. Impact of the antibiotic cocktail on GL261 viability *in vitro* and on gut functional parameters *in vivo*. (**A)** GL261 cells were grown in the presence of different concentrations of the same antibiotic cocktail as the one used *in vivo* for oral gavage. Cell viability was monitored using the MTT assay. 2 hours following MTT addition in the wells, cells were lysed in DMSO before recording the absorbance at 550 nm. (**B)** Fecal pellet output (FPO) of mice injected with GL261 or PBS and treated daily with antibiotics (ATB) or H_20. FPO was monitored before (d0) and 14 days (d14) after the surgery. Each dot represents one mouse that have been followed longitudinally between d0 and d14. Paired t test test was used to compare d0 and d14, $^*p<0.05$.
